# Supplementary material for: Complex‐centric proteome profiling by SEC‐SWATH‐MS
Source: Mol Syst Biol. 2019 Jan 14;15(1):e8438. doi: 10.15252/msb.20188438 (PMC6346213; doi:10.15252/msb.20188438)
Supplement: Supplementary file 8 — Dataset EV7 [file MSB-15-e8438-s008.zip › feature_plots_string/Q969L4.pdf]

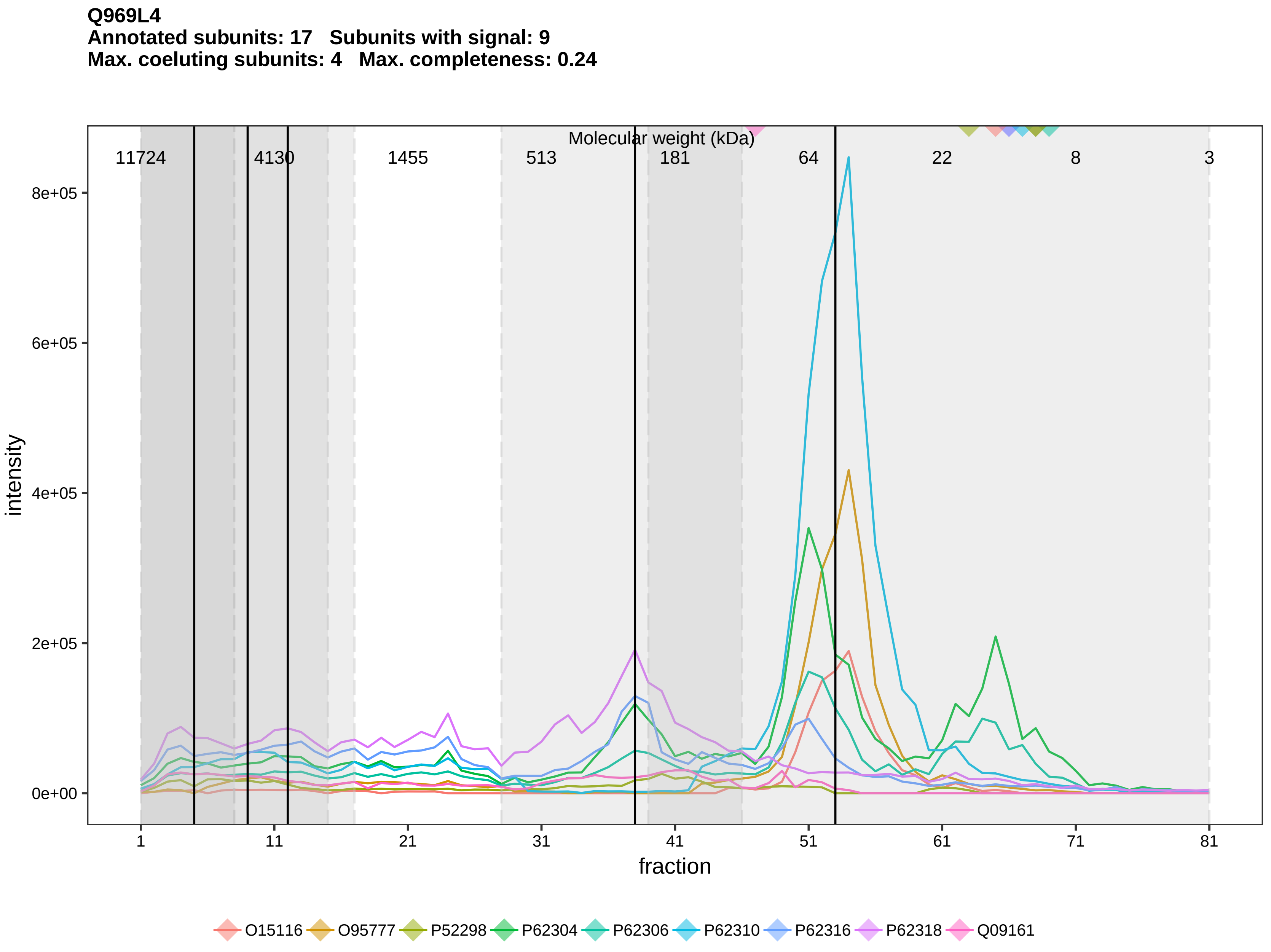
Q969L4
Annotated subunits: 17   Subunits with signal: 9
Max. coeluting subunits: 4   Max. completeness: 0.24
Molecular weight (kDa)
11724
4130
1455
513
181
64
22
8
3
intensity
8e+05
6e+05
4e+05
2e+05
0e+00
fraction
1
11
21
31
41
51
61
71
81
O15116
O95777
P52298
P62304
P62306
P62310
P62316
P62318
Q09161
